# Probiotic BC30 Improves Amino Acid Absorption from Plant Protein Concentrate in Older Women

**DOI:** 10.1007/s12602-022-10028-4

**Published:** 2022-12-14

**Authors:** Kylie E. Walden, Anthony M. Hagele, Logan S. Orr, Kristen N. Gross, Joesi M. Krieger, Ralf Jäger, Chad M. Kerksick

**Affiliations:** 1 Exercise and Performance Nutrition Laboratory, School of Health Sciences, Lindenwood University, St. Charles, MO USA; 2 Increnovo, LLC, Milwaukee, WI USA

**Keywords:** Probiotic, Amino acid, Protein, Plants, Aging, Absorption

## Abstract

*Weizmannia coagulans* GBI-30, 6086 (BC30) has previously been shown to increase protein digestion in an in vitro model of the stomach and small intestine and amino acid appearance in healthy men and women after ingestion of milk protein concentrate. The impact of ingesting BC30 with other protein sources or in other demographics is largely unknown. The purpose of this study was to examine the impact of adding BC30 to a 20-g dose of a blend of rice and pea protein on postprandial changes in blood amino acids concentrations in healthy, older women. Healthy, older females (*n* = 30, 58.5 ± 5.2 years, 165.4 ± 6.8 cm, 65.6 ± 8.8 kg, 23.7 ± 3.2 kg/m^2^) completed two separate 14-day supplementation protocols separated by a 3-week washout period. Participants were instructed to ingest a 20-g protein dose of a blend of rice and pea protein concentrates (ProDiem Plant Protein Solutions, Kerry) with (PPCBC30) or without (PPC) the addition of 1 × 10^9^ CFU BC30 (Kerry). Body composition and demographics were assessed upon arrival to the laboratory. Upon ingestion of their final assigned supplemental dose, blood samples were taken at 0 (baseline), 30-, 60-, 90-, 120-, 180-, and 240-min post-consumption and analyzed for amino acid concentrations. Alanine (*p* = 0.018), tryptophan (*p* = 0.003), cysteine (*p* = 0.041), essential amino acids (*p* = 0.050), and total amino acids (*p* = 0.039) all exhibited significantly (*p* ≤ 0.05) greater AUC with PPCBC30 when compared to PPC. In addition, tryptophan (*p* = 0.003), cysteine (*p* = 0.021), essential amino acids (*p* = 0.049), and total amino acids (*p* = 0.035) displayed significantly greater (*p* ≤ 0.05) concentration maximum (*C*_*Max*_) values in PPCBC30 when compared to PPC. Finally, time to reach *C*_*Max*_ (*T*_*Max*_) was similar between conditions with 80% of all measured amino acids and amino acid combinations achieving *C*_*Max*_ at a similar time (~ 60 min). Only phenylalanine *T*_*Max*_ was found to be different (*p* = 0.01) between the two conditions with PPC displaying a greater proportion of *T*_*Max*_ values after 30 min. Following qualitative (non-inferential) assessment, 88% of all measured outcomes achieved a higher AUC with PPCBC30 and 100% of all outcomes achieved a higher *C*_*Max*_ with PPCBC30. In concert with previous findings in a younger mixed gender cohort with milk protein, the addition of BC30 to a daily 20-g dose of plant protein concentrate in healthy older women improved AUC and *C*_*Max*_ values in several individual amino acids and amino acid combinations. Retrospectively registered on April 6, 2022, at ClinicalTrials.gov as NCT05313178.

## Introduction

In 2001, a group of international scientific experts working for the Food and Agriculture Organization of the United Nations (FAO) and World Health Organization (WHO) defined probiotics as “live microorganisms that, when administered in adequate amounts, confer a health benefit on the host” [1]. Established benefits from probiotic use include improvements in digestive and immune health [2]. Beyond these outcomes, probiotics can modulate pathogen adhesion and may augment the production of vitamins, short-chain fatty acids, and neurotransmitters [3]. The ability of probiotics to influence the absorption of key nutrients such as vitamins, minerals, carbohydrates, protein, and various forms of digestive enzymes has evolved into an emerging area of interest [4–6]. It is currently well-established that probiotic outcomes are strain specific. In particular, the *Weizmannia coagulans* GBI-30, 6086 (BC30) strain, formerly classified as *Bacillus coagulans*, is a lactic acid producing, spore-forming bacterial species that has exhibited the ability to improve protein and amino acid absorption [7, 8]. Largely due to its spore-forming ability, BC30 can withstand a range of temperatures and survive the harsh gut conditions while also demonstrating the ability to produce enzymes that can improve carbohydrate and protein digestion [9]. Functional outcomes associated with BC30 administration have included improvements in gastrointestinal symptoms and side effects such as abdominal pain and bloating [10, 11] in addition to facilitating the production of short-chain fatty acids. Short-chain fatty acid availability has been suggested by some to be critical to maintaining the health and vitality of the lining of the gut [12] while also exhibiting anti-inflammatory potential in several cell types found in the gut [13]. The ability to improve the gut lining and inflammation [13, 14] and the increase in the production of digestive enzymes are the candidate mechanistic links for BC30 to improve the absorption of amino acids into the bloodstream [7, 9] as well as improve protein digestion from both milk and plant proteins [15].

Recently, Stecker et al. [8] conducted a randomized, double-blind, crossover investigation in 30 healthy men and women (26.4 ± 6.5 years) to examine the impact of adding a 1 × 10^9^ CFU dose of BC30 to a 25-g dose of milk protein concentrate (20 g of protein delivered) on the appearance of amino acids into the bloodstream over a 4-h absorption time course. These authors concluded that area under the curve values for arginine and isoleucine were greater when BC30 was added to the milk protein dose. Additionally, greater maximum concentrations were also found for arginine, serine, ornithine, methionine, glutamic acid, phenylalanine, isoleucine, tyrosine, the sum of the essential amino acids, and the sum of all amino acids when BC30 was added to the milk protein dose. These results contrasted the results found by Townsend et al. [16] who conducted a similar study in 22 healthy active men (24.3 ± 3.2 years) that examined the ability of a different bacillus strain, *Bacillus subtilis* DE111, at a dose of 1 × 10^9^ CFU when combined with a 25-g dose of whey protein to impact amino acid appearance. These authors reported no differential responses between leucine, branched-chain amino acid, essential amino acids, and total amino acids and found no difference in area under the curve, highlighting that the benefits of probiotics are strain specific. Jäger and colleagues [6] published a similar investigation as the Stecker and Townsend papers whereby they also used 15 healthy, active men (24.2 ± 5.0 years) and had them co-ingest a 20-g dose of pea protein with and without the addition of a multi-strain probiotic (5 billion CFU L. *paracasei* LP-DG^®^ (CNCM I-1572) plus 5 billion CFU *L. paracasei* LPC-S01 (DSM 26760), SOFAR S.p.A., Italy) for two weeks. Amino acid appearance was compared, and the addition of probiotics increased the peak concentration and areas under the curve for several amino acids. The Jäger paper was significant as it was the first study to evaluate the impact of adding a probiotic to a plant protein source while all previous studies had utilized an animal protein source.

Plant proteins have become extremely popular for their potential health benefits in addition to offering a more environmentally sustainable protein source [17] but are often considered to be of a lower protein quality due to their lower protein and amino acid levels when compared to animal protein sources [18] in addition to them also exhibiting lower levels of digestibility [17]. Thus, it has been postulated that the addition of a probiotic to a plant protein may offer the ability to increase the digestive yield of the amino acids found within the protein source. While the Jäger paper offered insight into this potential outcome, more research is needed with other plant protein sources and probiotic strains to fully understand the potential.

In addition to protein source, the age of all recruited cohorts is another important consideration as it pertains to examining the potential impact of co-ingestion a probiotic with a protein source to improve amino acid appearance following digestion. As highlighted throughout, all previous studies [6, 8, 16] utilized cohorts of young participants ranging in average ages of 24–26 years of age. In this respect, “anabolic resistance” is a well-established phenomenon in the skeletal muscle of older adults whereby muscle protein synthesis is diminished in response to both resistance exercise [19] and protein ingestion [20, 21]. While greater doses of protein can somewhat overcome the established resistance [20], this recommendation may not be pragmatic as eating patterns of aging populations clearly indicate a reduced ability to consume enough dietary protein, much less the increased amounts that are commonly recommended for this population [22]. For these reasons, the need to examine the ability of co-ingesting a probiotic with dietary protein in an aging population is evident. The purpose of this study is to assess the impact of adding BC30 to a plant protein concentrate on amino acid appearance into the bloodstream in a cohort of healthy, older women.

## Methods

### Overview of Research Design

**Fig. 1:**
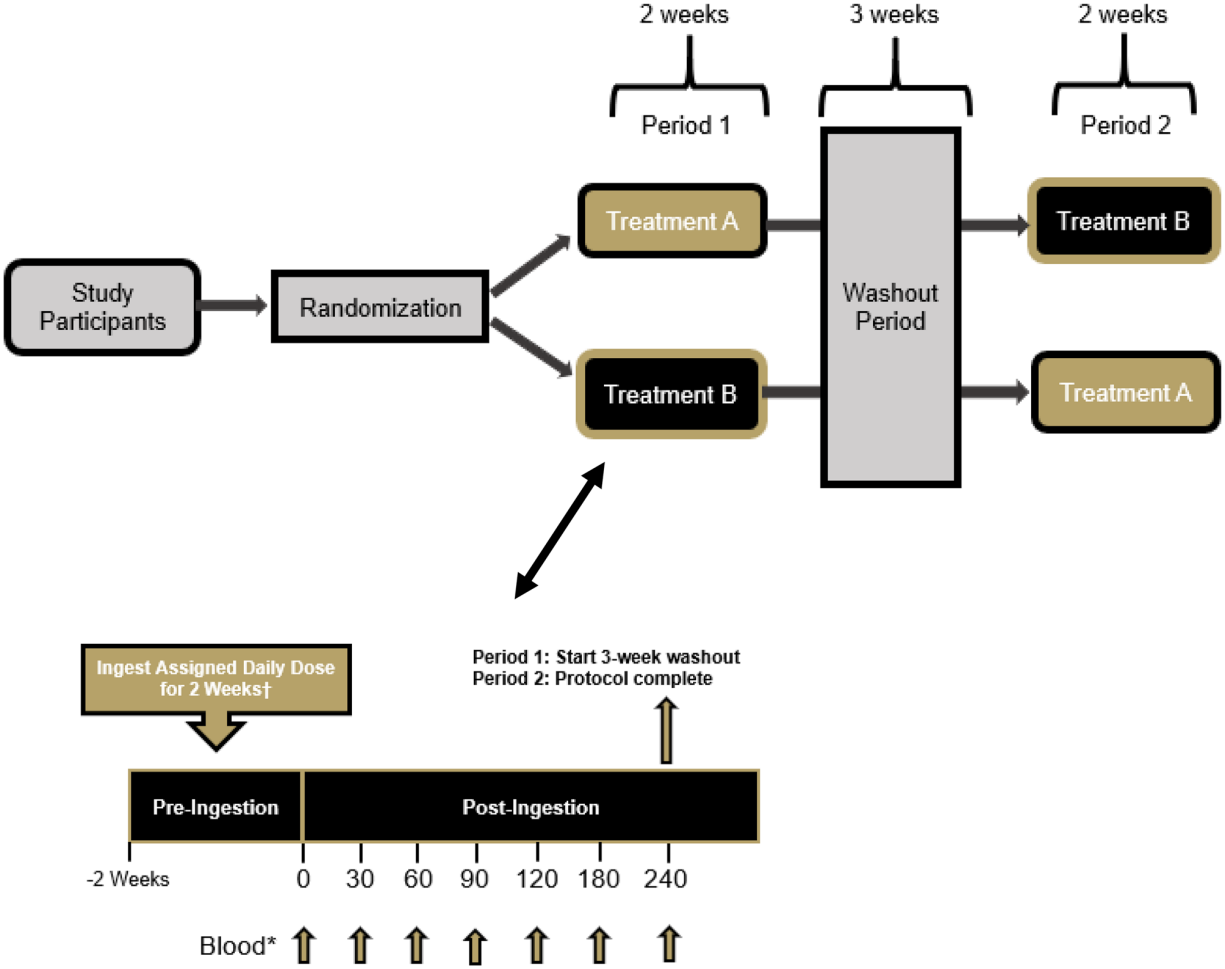
Overview of study design

In accordance with the methods of our previously published paper using an identical study design and assessment approach [8], this study was conducted using a randomized, double-blind, crossover study design. Healthy women (*n* = 30) between the ages of 50–70 years of age were recruited to participate in this study. Prior to beginning the study, all participants signed an IRB-approved informed consent document (Lindenwood University: IRB-21–57, approval date: 11/20/20) and completed a health history questionnaire to determine study eligibility. A priori sample size evaluation indicated that a sample size of 28–33 participants would be needed if an effect size of 0.5–0.55 was realized with an alpha (*α*) level of 0.05 and estimated power (1–β) of 0.80. This study protocol and design was retrospectively registered on Clinicaltrials.gov on April 6, 2022, as NCT05313178 (https://clinicaltrials.gov/ct2/show/NCT05313178). Two supplementation periods that each spanned 2 weeks were completed and separated with a washout period of 3 weeks (Fig. 1). For each study visit, all participants reported to the laboratory between 0600–1000 h after observing an 8 to 10 h fast. Prior to each study visit, participants were assigned to ingest 13 daily doses of a pea and rice protein blend with or without the inclusion of a 1 × 10^9^ colony forming units (CFU) dose of BC30. To minimize any order effects from testing, participants were randomized using an online randomization software program (www.random.org). Upon arrival for each study visit, participants had their height, body mass, body composition, and hemodynamics assessed. A series of venous blood collections were then collected. After collection of the first (0 min) blood sample, study participants ingested the 14th and final dose of their assigned supplement before having subsequent venous blood samples (~ 10 mL) collected 30, 60, 90, 120, 180, and 240 min after ingestion (Fig. 1). Participants were provided 200 mL of cold water to ingest after each blood collection. Upon processing, all blood samples were stored at − 80 °C. Prior to leaving, study participants were provided all doses of the alternative treatment to begin after observing a 3-week washout. After consuming 13 consecutive doses of the next assigned study treatment, participants returned to the laboratory for their remaining testing visit, which was completed in an identical fashion.

### Study Participants

**Fig. 2:**
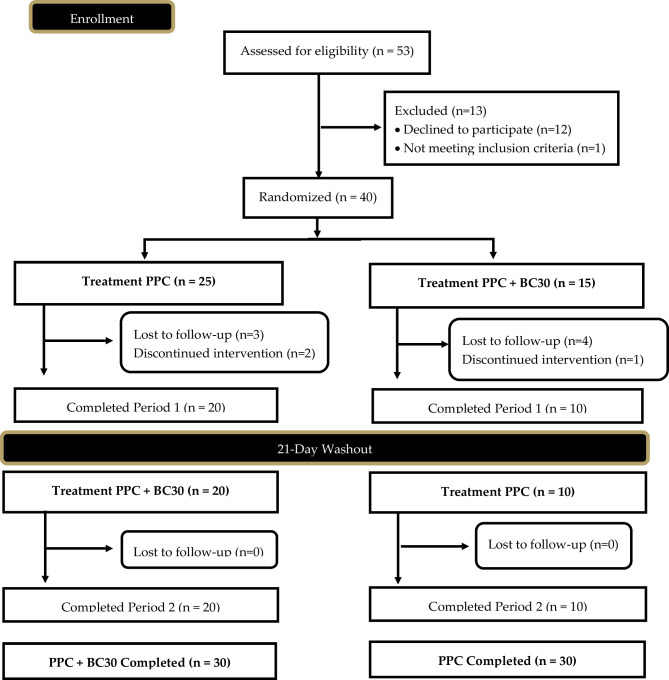
CONSORT diagram

In total, 30 females (58.5 ± 5.2 years, 165.4 ± 6.8 cm, 65.6 ± 8.8 kg, 23.7 ± 3.2 kg/m^2^, 31.2 ± 9.1% fat) successfully completed all study visits (see Table 1). A Consolidation Standards of Reporting Trials (CONSORT) diagram was created to examine all study recruitment, randomization methods, and project completion and is provided in Fig. 2. Inclusion criteria included age (50–70 years), healthy and free of disease (as reported by the health screening questionnaire), and physically active (reported at least 30 min of moderate exercise three days a week). Any individual diagnosed with or being treated for cardiac, respiratory, circulatory, musculoskeletal, metabolic, obesity (defined as body mass index > 30 kg/m^2^ and body fat greater than 30%), immune, autoimmune, psychiatric, hematological, neurological, or endocrinological disorder or disease was not allowed to participate in the current study (Table 2).

**Table 1:** Baseline age, gender, height (cm), weight (kg), body mass index, % fat, heart rate, systolic blood pressure, diastolic blood pressure, energy, carbohydrates, proteins, and fat intake

*N* = 30	Mean	SD
Age	58.5	5.2
Height (cm)	165.4	6.8
Weight (kg)	65.6	8.8
Body mass index (kg/m_2_)	23.7	3.2
% fat	31.2	9.1
Heart rate (beats/min)	64.8	7.9
Systolic blood Pressure (mm Hg)	112.0	18.7
Diastolic blood pressure (mm Hg)	70.6	11.7
Energy intake (kcals/day)	1668	428
Carbohydrate intake (grams/day)	164	65
Protein intake (grams/day)	68	24
Fat intake (grams/day)	94	26

**Table 2:** Composition of ingested proteins. Analysis completed by Eurofins, Madison, WI, by HPLC. Report 3710869–0 (June 21, 2022) and 37093130–0 (June 20, 2022). A 27-g serving was administered to each participant for daily administration. Total nitrogen content was analyzed by the Dumas method and was determined to be 3.32-g protein in a 27-g dose. The protein content of the plant protein concentrate was determined to be 76.8% protein

	**PPC**		**PPCBC30**	
	**AA (mg/serving)**	**AA (mg/g protein)**	**AA (mg/serving)**	**AA (mg/g protein)**
Aspartic acid	2182	105.3	2182	105.3
Threonine	733	35.4	726	35.0
Serine	1020	49.2	1017	49.1
Glutamic acid	3305	159.5	3272	157.9
Proline	870	42.0	867	41.9
Glycine	820	39.6	818	39.5
Alanine	951	45.9	933	45.0
Valine	1044	50.4	1041	50.2
Isoleucine	912	44.0	922	44.5
Leucine	1652	79.7	1652	79.7
Tyrosine	848	40.9	837	40.4
Phenylalanine	1080	52.1	1091	52.6
Lysine	1210	58.4	1231	59.4
Histidine	440	21.2	444	21.4
Arginine	1652	79.7	1663	80.3
Cystine	232	11.2	222	10.7
Methionine	305	14.7	302	14.6
Tryptophan	202	9.7	200	9.6
**Total (mg)**	19,457	938.9	19,422	937.2

### Procedures

#### Baseline Demographics and Hemodynamics

Notwithstanding the differences in age, gender, and the protein source delivered, the completed methods for the present study were designed to be identical to the procedures outlined in Stecker et al. [8]. During the initial visit, after providing written consent, participants were instructed to rest quietly for approximately 10 min before measuring resting heart rate and blood pressure (Omron BP785, Omron Corporation, Kyoto, Japan). Participants then had their body mass determined (Tanita BWB-627A, Tokyo, Japan) and recorded to the nearest ± 0.1 kg upon arrival prior to beginning each study visit. All recorded body masses were compared to ensure the participant was weight stable. Any participant whose body mass changed by more than 2% between consecutive study visits was excluded from participation. Following body mass measurements, the height was measured using a standard wall-mounted stadiometer (Tanita, HR-200, Tokyo, Japan) and recorded to the nearest ± 0.5 cm. Fat and fat-free mass was determined using a bioelectrical impedance analyzer (InBody 570, Beverly Hills, California). To stabilize body composition assessments as well as any potential diurnal changes in primary endpoints, study participants were required to observe an overnight fast, and all testing visits were scheduled at near identical times with all visits commencing between 0600 and 1000 h.

#### Dietary Monitoring

Prior to their baseline visit, study participants completed a hand-written 4-day food record (three weekdays and one weekend day). A copy of the 4-day food log was made and provided to study participants to facilitate diet replication for the subsequent study period. Food records were analyzed using MyFitnessPal (San Francisco, CA). Average energy, carbohydrate, fat, and protein intake were recorded.

#### Venous Blood Collection and Processing

During two separate identical study visits, study participants had an indwelling catheter implanted or single stick venipuncture completed using a forearm vein to repeatedly sample venous blood. A total of seven venous blood samples were collected (Fig. 2) into two ethylenediaminetetraacetic acid (EDTA) Vacutainer™ tubes at 0, 30, 60, 90, 120, 180, and 240 min for each of the participant’s study visits. For each collection, tubes were gently inverted 10 times before being centrifuged at 4 °C for 20 min at 2,000 revolutions per minute (rpm) (MegaFuge XFR, Thermo Fisher Scientific, Waltham, MA, USA). After the completion of centrifuging, plasma was aliquoted (~ 600µL) into separate micro-centrifuge tubes and appropriately labeled with subject identification, condition, and time-point. Once the samples were aliquoted into their respective microcentrifuge tubes, they were stored at − 80 °C for later amino acid analysis.

#### Supplementation Protocol

In a randomized, double-blind, crossover fashion, study participants supplemented daily for 2 weeks during each study period with a 27-g dose of a rice and pea protein concentrate blend (ProDiem Plant Protein Solutions, Kerry, Beloit, WI, USA) which yielded a 20-g dose of protein. During one supplementation period 1 × 10^9^ colony forming units (CFU) dose of BC30 in the spore form (Kerry, Beloit, WI, USA) was added to each protein dose. An equivalent mass of maltodextrin (Kerry, Beloit, WI, USA) was added during the alternative supplementation condition, which served as the placebo arm of the study. All study materials were blinded by weighing out the required amounts into individual plastic sachets and labeled with non-identifying numbers and letters. All protein doses were measured prior to supplementation using a calibrated analytical balance before adding appropriate doses of maltodextrin and BC30 to each sachet. Each sachet was thoroughly mixed to prevent any ability to decipher between two products. Participants were instructed to consume each dose at approximately the same time of day with 237–355 mL of cold tap water. All participants were required to complete a supplementation log to document when each dose of their assigned protein was consumed. Upon completion of their first assigned study protocol period, participants observed a 3-week washout period by returning to their normal dietary intake and physical activity habits before beginning supplementation for the second study period. Protein content and amino acid composition was verified independently by Eurofins, Madison, WI, USA, after the completion of the study.

#### Amino Acid Determination

Amino acid analysis was performed by Heartland Assays (Iowa State University Research Park, Ames, IA, USA). Plasma samples were assayed for the concentration of 20 different amino acids (arginine, glutamine, citrulline, serine, asparagine, glycine, threonine, alanine, ornithine, methionine, proline, lysine, aspartic acid, histidine, valine, glutamic acid, tryptophan, leucine, phenylalanine, isoleucine, cystine, tyrosine) using a standardized liquid chromatography, mass spectrometry procedure. Briefly, EZ:faast® amino acid analysis kits (Phenomenex, Torrance, CA) were used for liquid chromatographic analysis of amino acids using tandem-mass spectrometry (LC/MS/MS) and electrospray ionization (ESI) (Agilent, Santa Clara, CA). The procedure consisted of solid phase extraction of 25 µl of plasma with internal standards by a sorbent tip attached to a syringe with an eluting solvent (a 3:2 mixture of sodium hydroxide with 77% n-propanol, and 23% 3-picoline). The free amino acids were then derivatized by adding a mixture of 17.4% propyl chloroformate, 11% isooctane, and 71.6% chloroform. The resulting mixture was vortexed and allowed to sit at room temperature for 1 min, followed by liquid–liquid extraction with isooctane. The organic layer was removed, dried under nitrogen gas, and suspended in the HPLC run solvents before being injected into the LC/MS/MS. Chromatographic separation of the derivatized amino acids was conducted on an EZ:faast amino acid analysis-mass spectrometry column (250 × 2.0 mm i.d., 4 µm) using a Agilent 6460 triple quadrupole LC/MS/MS system (Santa Clara, CA). Next, 10-mM ammonium formate in water with 0.2% formic acid (mobile phase A) and 10-mM ammonium formate in methanol with 0.2% formic acid (mobile phase B) were used as the solvent system with gradient conditions of 68% of mobile phase B at 0 min to 83% mobile phase B over 13 min with a flow rate of 0.25 ml/min. Amino acids and internal standard data were collected using the dynamic multiple reaction monitoring mode using MassHunter acquisition software (Agilent, Santa Clara, CA). MassHunter Quantitation software was used to quantitate the unknown plasma samples based on best fit standard curves.

#### Adverse Event Reporting

Study participants were asked to verbally report the incidence and severity of any adverse events (dizziness, headache, nausea, upset stomach, cramping, diarrhea, etc.) throughout consumption of either test product.

### Statistical Analysis

Primary outcomes for this trial were considered to be area under the curve (AUC) data for the measured amino acids. Secondary outcomes were considered to the maximum concentrations (*C*_*Max*_) identified for the measured amino acids. All analyses were completed using Microsoft Excel and the Statistical Package for the Social Sciences (v23; SPSS Inc., Chicago IL). For all dependent measures, descriptive statistics were presented as mean ± standard deviations. Before any statistical tests were completed, the normality was assessed for all dependent variables. All reported *p* values are computed using parametric approaches. Paired sample *t* tests were completed to determine between-group differences for the AUC and *C*_*Max*_ values for all individually measured amino acids as well as the sum of the branched-chain (BCAA), essential (EAA), and total (TAA) amino acids. For all statistical tests, data was considered statistically significant when the probability of type I error was 0.05 or less. Between-group effect sizes, *p* values and 95% confidence intervals were computed and are provided in the tables.

## Results

### Dietary Replication and Hemodynamics

Study participants reported 100% compliance to completing the 4-day food record. Dietary intake data is provided in Table 1. In addition, resting heart rate and blood pressure are provided in Table 1.

### Supplementation Compliance

Compliance to each supplementation period was very good. A total of four doses were reported as being missed during the PPC condition. In detail, no more than one dose was missed by any given participant. One dose was missed by four different participants. Thus, 26 people reported 100% compliance while four people reported 92.8% compliance resulting in an overall compliance during PPC of 99.04%. Similarly, a total of four doses were reported as being missed during the PPCBC30 condition. One dose was missed by four different participants. Thus, 26 people reported 100% compliance while four people reported 92.8% compliance resulting in an overall compliance during PPC of 99.04%. Thus, overall compliance to the supplementation regimen was 99.04%.

### Blinding Efficacy

To assess how well each supplement was blinded, we asked each participant which supplement condition they thought they were assigned when the final blood sample was collected. During this first assigned study period, 16/30 (53.3%) participants reported not knowing which condition they were assigned. Of the remaining who did indicate one condition or the other, 7/30 (23.3%) participants incorrectly identified their assigned supplement condition, and an additional 7/30 (23.3%) participants correctly identified their assigned group. Overall, 76.6% of the participants either didn’t know or incorrectly guessed their assigned supplement condition during study period 1 while 23.3% of participants correctly guessed their assigned supplement. During this second assigned study period, 14/30 (46.7%) participants reported not knowing which condition they were assigned. Of the remaining who did indicate one condition or the other, 8/30 (26.7%) participants incorrectly identified their assigned supplement condition, and an additional 8/30 (26.7%) participants correctly identified their assigned group. Overall, 73.3% of the participants either didn’t know or incorrectly guessed their assigned supplement condition during study period 2 while 26.7% of participants correctly guessed their assigned supplement. Consequently, the blinding was deemed a success for this study trial.

### Amino Acids Area Under the Curve (AUC, µmol/L • 180 min)

Significantly greater area under the curves were observed when BC30 was added to the plant protein blend for alanine (PPC: 807 ± 166 µmol/L vs. PPCBC30: 856 ± 172 µmol/L, *p* = 0.018, 6.0% difference, *d* = 0.29, (95% CI: 9.1, 88.5 µmol/L • 180 min), ornithine (PPC: 201 ± 59 µmol/L vs. PPCBC30: 216 ± 63 µmol/L, *p* = 0.05, 7.7% difference, *d* = 0.25, (95% CI: − 0.26, 31.4 µmol/L • 180 min), tryptophan (PPC: 164 ± 48 µmol/L vs. PPCBC30: 177 ± 44 µmol/L, *p* = 0.003, 8.0% difference, *d* = 0.29, (95% CI: 4.8, 21.4 µmol/L • 180 min), phenylalanine (PPC: 160 ± 30 µmol/L vs. PPCBC30: 165 ± 28 µmol/L, *p* = 0.053, 3.1% difference, *d* = 0.05, (95% CI: -0.06, 9.8 µmol/L • 180 min), cysteine (PPC: 112 ± 23 µmol/L vs. PPCBC30: 117 ± 19 µmol/L, *p* = 0.04, 4.7% difference, *d* = 0.25 (95% CI: 0.22, 10.2 µmol/L • 180 min), total essential amino acids (PPC: 2667 ± 436 µmol/L vs. PPCBC30: 2785 ± 384 µmol/L, *p* = 0.05, 4.4% difference, *d* = 0.29, (95% CI: 0.26, 237 µmol/L • 180 min), and total amino acids (PPC: 9069 ± 1184 µmol/L vs. PPCBC30: 9409 ± 1341 µmol/L, *p* = 0.04, 3.8% difference, *d* = 0.27, (95% CI: 18.3, 662.9 µmol/L • 180 min) (Table 3).

**Table 3:** Area under the curve (AUC) values for each individual amino acid, total BCAA, total EAA, and total amino acids

	**PPCBC30**		**PPC**		**PPCBC30 vs. PPC**			
**Amino acid**	**Mean**	**SD**	**Mean**	**SD**	***p* value (*t* test)**	**ES (d)**	**%**	**95% CI**
**Arginine**	373	94	360	82	0.241	0.15	3.6	(-9.2, 34.9)
**Glutamine**	2352	411	2318	348	0.267	0.09	1.5	(-28.1, 97.8)
**Citrulline**	101.4	32.6	102.0	36.3	0.851	-0.02	-0.6	(-7.0, 5.8)
**Serine**	303.8	78.6	291.3	61.7	0.157	0.18	4.3	(-5.1, 30.0)
**Asparagine**	418.1	143.4	419.7	125.1	0.904	-0.01	-0.4	(-28.4, 25.2)
**Glycine**	751.9	297	698.5	185.1	0.174	0.22	7.6	(-25.0, 131.7)
**Threonine**	359.6	82.3	351.4	76.0	0.404	0.10	2.3	(-11.6, 28.0)
**Alanine**	856.0^a^	172.0	807.2	166.3	0.018	0.29	6.0	(9.1, 88.5)
**Ornithine**	216.3^a^	63.0	200.8	58.8	0.054	0.25	7.7	(-0.26, 31.4)
**Methionine**	75.4	15.7	73.5	15.5	0.349	0.12	2.6	(-2.2, 5.9)
**Proline**	420.5	130.3	390.5	116.4	0.100	0.24	7.7	(-6.1, 66.1)
**Lysine**	603.9	96.8	581.5	117.0	0.093	0.21	3.9	(-4.0, 48.8)
**Aspartic acid**	13.9	5.2	12.6	3.5	0.082	0.29	10.3	(-0.17, 2.7)
**Histidine**	198.4	33.1	191.9	31.8	0.089	0.20	3.4	(-1.1, 14.1)
**Valine**	372.7	93.6	359.8	81.8	0.241	0.15	3.6	(-9.2, 34.9)
**Glutamic acid**	109.2	37.0	106.2	40.7	0.612	0.08	2.8	(-9.0, 15.0)
**Tryptophan**	176.6^a^	43.6	163.5	47.5	0.003	0.29	8.0	(4.8, 21.4)
**Leucine**	465.7	83.6	444.4	87.5	0.073	0.25	4.8	(-2.1, 44.6)
**Phenylalanine**	164.6^a^	28.2	159.7	29.7	0.053	0.17	3.1	(-0.06, 9.8)
**Isoleucine**	260.0	51.6	248.3	55.9	0.124	0.22	4.7	(-3.4, 26.6)
**Cysteine**	116.7^a^	19.4	111.5	22.8	0.041	0.25	4.7	(0.22, 10.2)
**Tyrosine**	231.4	52.5	232.8	62.2	0.852	-0.02	-0.6	(-15.7, 13.1)
**Total BCAA**	1566.2	255.8	1496.4	274.1	0.103	0.26	4.7	(-15.1, 154.8)
**Total EAA**	2785.1^a^	384.3	2666.7	435.5	0.050	0.29	4.4	(0.26, 236.5)
**Total amino acids**	9409.3^a^	1340.6	9068.7	1183.5	0.039	0.27	3.8	(18.3, 662.9)

Values inside table are the calculated areas under the curves (AUC) using the trapezoidal rule. Unit of measure for AUC is μmol /L • 180 min

^a^Between-group difference (*p* < 0.05) using paired samples *t* test. % = Percent difference between groups

**Table 4:** Maximum observed concentration (*C*_*Max*_) for each individual amino acid, total BCAA, total EAA, and total amino acids

	**PPC + BC30**		**PPC**		**PPC + BC30 vs. PPC**			
**Amino acid**	**Mean**	**SD**	**Mean**	**SD**	***p* value (*t* test)**	**ES (d)**	**%**	**95% CI**
**Arginine**	147.3	42.5	139.7	39.3	0.258	0.19	5.4	(− 5.9, 21.1)
**Glutamine**	670.0	123.2	655.7	105.2	0.225	0.12	2.2	(− 9.3, 38.0)
**Citrulline**	32.4	8.5	31.7	9.2	0.586	0.08	2.2	(− 1.9, 3.2)
**Serine**	100.7	26.1	96.9	20.7	0.257	0.16	3.9	(− 2.9, 10.6)
**Asparagine**	157.4	59.7	151.7	49.8	0.367	0.10	3.8	(− 7.1, 18.5)
**Glycine**	224.2	83.9	208.8	52.5	0.160	0.22	7.4	(− 6.4, 37.3)
**Threonine**	114.4	27.0	111.2	23.5	0.339	0.13	2.9	(− 3.6, 10.0)
**Alanine**	267.7	59.0	251.3	47.0	0.054	0.31	6.5	(− 0.27, 32.9)
**Ornithine**	72.1	22.9	69.7	23.7	0.493	0.10	3.4	(− 4.8, 9.7)
**Methionine**	23.7	5.2	23.5	5.7	0.672	0.04	0.9	(− 1.0, 1.5)
**Proline**	135.6	37.5	125.7	31.5	0.069	0.29	7.9	(− 0.80, 20.4)
**Lysine**	213.1	31.8	203.5	33.8	0.066	0.29	4.7	(− 0.67, 20.0)
**Aspartic acid**	5.8	2.9	5.0	1.9	0.125	0.33	16.0	(− 0.24, 1.8)
**Histidine**	62.7	14.4	60.7	17.2	0.553	0.13	3.3	(− 4.7, 8.5)
**Valine**	147.3	42.5	139.7	39.3	0.258	0.19	5.4	(− 5.9, 21.1)
**Glutamic Acid**	42.4	16.9	40.5	16.7	0.550	0.11	4.7	(− 4.5, 8.3)
**Tryptophan**	62.0^a^	16.7	55.1	14.8	0.003	0.44	12.5	(2.5, 11.3)
**Leucine**	172.7	27.0	165.6	25.2	0.170	0.27	4.3	(− 3.2, 17.5)
**Phenylalanine**	52.4	7.6	51.5	8.2	0.510	0.11	1.7	(− 1.8, 3.5)
**Isoleucine**	100.4	15.2	95.6	15.8	0.148	0.31	5.0	(− 1.8, 11.4)
**Cysteine**	32.9^a^	5.2	31.2	6.0	0.021	0.30	5.4	(0.28, 3.2)
**Tyrosine**	77.4	15.7	77.0	16.1	0.856	0.03	0.5	(− 4.2, 5.0)
**Total BCAA**	537.3	76.1	510.5	72.9	0.083	0.36	5.2	(− 3.7, 57.3)
**Total EAA**	940.2^a^	115.7	893.2	120.4	0.049	0.40	5.3	(0.17, 93.9)
**Total** **amino acids**	2969.6^a^	423.4	2840.6	358.9	0.035	0.33	4.5	(10.0, 248.2)

Data provided is means ± SD. *C*_*Max*_ = Maximum observed concentration (in μmol/L) for each condition

^a^Between-group difference (*p* < 0.05) using paired samples *t* test. % = Percent difference between groups

### Maximum Amino Acid Concentration (C_MAX_, µmol/L)

As seen in Table 4, Significantly greater maximum concentrations were observed when BC30 was added to the plant protein blend for tryptophan (PPC: 55.1 ± 14.8 µmol/L vs. PPCBC30: 62.0 ± 16.7 µmol/L, *p* = 0.003, 12.5% difference, *d* = 0.44, (95% CI: 2.5, 11.3 µmol/L), cysteine (PPC: 31.2 ± 6.0 µmol/L vs. PPCBC30: 32.9 ± 5.2 µmol/L, *p* = 0.02, 5.4% difference, *d* = 0.30, (95% CI: 0.28, 3.2 µmol/L), total essential amino acids (PPC: 893 ± 120 µmol/L vs. PPCBC30: 940 ± 116 µmol/L, *p* = 0.05, 5.3% difference, *d* = 0.40, (95% CI: 0.17, 93.9 µmol/L), and total amino acids (PPC: 2841 ± 359 µmol/L vs. PPCBC30: 2970 ± 423 µmol/L, *p* = 0.04, 4.5% difference, *d* = 0.33, (95% CI: 10.0, 248.2 µmol/L).

### Measured Time Point for Maximum Amino Acid Concentration (T_*Max*_)

As seen in Table 5, the time to maximum concentration for phenylalanine concentrations was different (*p* = 0.01) between PPC (median = 60 min; IQR = 52.5–60 min) and PPCBC30 (median = 60 min, IQR = 30–60 min). Additionally, the time to maximum concentration for isoleucine concentrations tended to be different (*p* = 0.08) between PPC (median = 60 min; IQR = 60–60 min) and PPCBC30 (median = 60 min, IQR = 30–60 min). All other measured *T*_*Max*_ values were not different between conditions.

**Table 5:** Time (minutes) to maximum concentration (*T*_*Max*_): individual amino acids, total BCAA, total EAA, and total amino acid

**Amino acid**	**Group**	**0**	**30**	**60**	**90**	**120**	**180**	240	*p*
**Arginine**	PPC + BC30	0 (0.0%)	12 (40.0%)	17 (56.7%)	1 (3.3%)	0 (0.0%)	0 (0.0%)	0 (0.0%)	0.19
	PPC	0 (0.0%)	11 (36.7%)	13 (43.3%)	5 (16.7%)	1 (3.3%)	0 (0.0%)	0 (0.0%)	
**Glutamine**	PPC + BC30	0 (0.0%)	5 (16.7%)	21 (70.0%)	4 (13.3%)	0 (0.0%)	0 (0.0%)	0 (0.0%)	0.76
	PPC	1 (3.3%)	9 (30.0%)	13 (43.3%)	5 (16.7%)	0 (0%)	1 (3.3%)	1 (3.3%)	
**Citrulline**	PPC + BC30	6 (24.0%)	3 (12.0%)	9 (36.0%)	2 (8.0%)	3 (12.0%)	2 (8.0%)	0 (0.0%)	0.45
	PPC	9 (30.0%)	3 (10.0%)	7 (23.3%)	1 (3.3%)	3 (10.0%)	5 (16.7%)	2 (6.7%)	
**Serine**	PPC + BC30	1 (3.3%)	8 (26.7%)	20 (66.7%)	0 (0.0%)	1 (3.3%)	0 (0.0%)	0 (0.0%)	0.51
	PPC	1 (3.3%)	13 (43.3%)	14 (46.7%)	2 (6.7%)	0 (0.0%)	0 (0.0%)	0 (0.0%)	
**Asparagine**	PPC + BC30	0 (0.0%)	8 (26.7%)	22 (73.3%)	0 (0.0%)	0 (0.0%)	0 (0.0%)	0 (0.0%)	0.49
	PPC	0 (0.0%)	14 (46.7%)	13 (43.3%)	2 (6.7%)	1 (3.3%)	0 (0.0%)	0 (0.0%)	
**Glycine**	PPC + BC30	2 (6.9%)	7 (24.1%)	19 (65.5%)	0 (0.0%)	1 (3.4%)	0 (0.0%)	0 (0.0%)	0.62
	PPC	2 (6.7%)	14 (46.7%)	10 (33.3%)	2 (6.7%)	2 (6.7%)	0 (0.0%)	0 (0.0%)	
**Threonine**	PPC + BC30	0 (0.0%)	5 (17.2%)	23 (79.3%)	1 (3.4%)	0 (0.0%)	0 (0.0%)	0 (0.0%)	0.22
	PPC	0 (0.0%)	12 (40.0%)	14 (46.7%)	3 (10.0%)	1 (3.3%)	0 (0.0%)	0 (0.0%)	
**Alanine**	PPC + BC30	0 (0.0%)	5 (16.7%)	20 (66.7%)	4 (13.3%)	1 (3.3%)	0 (0.0%)	0 (0.0%)	0.45
	PPC	0 (0.0%)	3 (10.0%)	21 (70.0%)	5 (16.7%)	1 (3.3%)	0 (0.0%)	0 (0.0%)	
**Ornithine**	PPC + BC30	0 (0.0%)	2 (6.7%)	18 (60.0%)	5 (16.7%)	4 (13.3%)	1 (3.3%)	0 (0.0%)	0.81
	PPC	0 (0.0%)	5 (16.7%)	13 (43.3%)	7 (23.3%)	2 (6.7%)	3 (10.0)	0 (0.0%)	
**Methionine**	PPC + BC30	0 (0.0%)	12 (40.0%)	15 (50.0%)	2 (6.7%)	1 (3.3%)	0 (0.0%)	0 (0.0%)	0.48
	PPC	0 (0.0%)	13 (43.3%)	15 (50.0%)	2 (6.7%)	0 (0.0%)	0 (0.0%)	0 (0.0%)	
**Proline**	PPC + BC30	0 (0.0%)	5 (17.2%)	22 (75.9%)	1 (3.4%)	1 (3.4%)	0 (0.0%)	0 (0.0%)	0.56
	PPC	0 (0.0%)	10 (33.3%)	15 (50.0%)	3 (10.0%)	2 (6.7%)	0 (0.0%)	0 (0.0%)	
**Lysine**	PPC + BC30	1 (3.3%)	4 (13.3%)	24 (80.0%)	1 (3.3%)	0 (0.0%)	0 (0.0%)	0 (0.0%)	0.56
	PPC	0 (0.0%)	11 (36.7%)	16 (53.3%)	2 (6.7%)	1 (3.3%)	0 (0.0%)	0 (0.0%)	
**Aspartic acid**	PPC + BC30	1 (3.3%)	15 (50.0%)	9 (30.0%)	2 (6.7%)	2 (6.7%)	1 (3.3%)	0 (0.0%)	0.82
	PPC	5 (16.7%)	11 (36.7%)	8 (26.7%)	2 (6.7%)	2 (6.7%)	2 (6.7%)	0 (0.0%)	
**Histidine**	PPC + BC30	0 (0.0%)	6 (20.0%)	24 (80.0%)	0 (0.0%)	0 (0.0%)	0 (0.0%)	0 (0.0%)	0.36
	PPC	0 (0.0%)	13 (43.3%)	15 (50.0%)	0 (0.0%)	2 (6.7%)	0 (0.0%)	0 (0.0%)	
**Valine**	PPC + BC30	0 (0.0%)	12 (40.0%)	17 (56.7%)	1 (3.3%)	0 (0.0%)	0 (0.0%)	0 (0.0%)	0.19
	PPC	0 (0.0%)	11 (36.7%)	13 (43.3%)	5 (16.7%)	1 (3.3%)	0 (0.0%)	0 (0.0%)	
**Glutamic acid**	PPC + BC30	4 (13.3%)	10 (33.3%)	10 (33.3%)	3 (10.0%)	0 (0.0%)	3 (6.7%)	0 (0.0%)	0.93
	PPC	6 (20.0%)	9 (30.0%)	9 (30.0%)	3 (10.0%)	1 (3.3%)	1 (3.3%)	1 (3.3%)	
**Tryptophan**	PPC + BC30	1 (3.3%)	7 (24.1%)	14 (48.3%)	4 (13.8%)	2 (6.9%)	1 (3.4%)	0 (0.0%)	0.30
	PPC	1 (3.3%)	12 (40.0%)	9 (30.0%)	5 (16.7%)	2 (6.7%)	1 (3.3%)	0 (0.0%)	
**Leucine**	PPC + BC30	0 (0.0%)	7 (24.1%)	22 (75.9%)	0 (0.0%)	0 (0.0%)	0 (0.0%)	0 (0.0%)	0.34
	PPC	0 (0.0%)	13 (43.3%)	14 (46.7%)	1 (3.3%)	1 (3.3%)	1 (3.3%)	0 (0.0%)	
**Phenylalanine**	PPC + BC30	0 (0.0%)	7 (24.1%)	17 (58.6%)	3 (10.3%)	1 (3.4%)	1 (3.4%)	0 (0.0%)	0.01
	PPC	0 (0.0%)	13 (43.3%)	14 (46.7%)	3 (10.0%)	0 (0.0%)	0 (0.0%)	0 (0.0%)	
**Isoleucine**	PPC + BC30	0 (0.0%)	5 (17.2%)	22 (75.9%)	2 (6.9%)	0 (0.0%)	0 (0.0%)	0 (0.0%)	0.08
	PPC	0 (0.0%)	12 (40.0%)	16 (53.3%)	2 (6.7%)	0 (0.0%)	0 (0.0%)	0 (0.0%)	
**Cysteine**	PPC + BC30	6 (20.0%)	6 (20.0%)	15 (50.0%)	3 (10.0%)	0 (0.0%)	0 (0.0%)	0 (0.0%)	0.83
	PPC	4 (13.3%)	14 (46.7%)	9 (30.0%)	2 (6.7%)	1 (3.3%)	0 (0.0%)	0 (0.0%)	
**Tyrosine**	PPC + BC30	0 (0.0%)	4 (13.8%)	22 (75.9%)	2 (6.9%)	1 (3.4%)	0 (0.0%)	0 (0.0%)	0.51
	PPC	0 (0.0%)	5 (16.7%)	17 (56.7%)	4 (13.3%)	3 (10.0%)	1 (3.3%)	0 (0.0%)	
**Total BCAA**	PPC + BC30	0 (0.0%)	4 (13.8%)	22 (75.9%)	2 (6.9%)	1 (3.4%)	0 (0.0%)	0 (0.0%)	0.33
	PPC	0 (0.0%)	8 (26.7%)	17 (56.7%)	2 (6.7%)	2 (6.7%)	1 (3.3%)	0 (0.0%)	
**Total EAA**	PPC + BC30	0 (0.0%)	5 (17.2%)	22 (75.9%)	2 (6.9%)	0 (0.0%)	0 (0.0%)	0 (0.0%)	0.15
	PPC	0 (0.0%)	11 (36.7%)	16 (53.3%)	2 (6.7%)	1 (3.3%)	0 (0.0%)	0 (0.0%)	
**Total amino acids**	PPC + BC30	0 (0.0%)	4 (13.3%)	25 (83.3%)	1 (3.3%)	0 (0.0%)	0 (0.0%)	0 (0.0%)	0.41
	PPC	0 (0.0%)	11 (36.7%)	15 (50.0%)	3 (10.0%)	1 (3.3%)	0 (0.0%)	0 (0.0%)	

Data is presented as number of times the specified timepoint was *T*_*Max*_. The value in parenthesis is the % occurrence within the condition for each timepoint. *T*_*Max*_ = Timepoint (in minutes) at which maximum concentration was observed. *p* = difference determined using Wilcoxon signed-rank test

### Adverse Event Reporting

Both PPC and PPCBC30 were well tolerated. A total of six adverse events were reported during the PPC condition ranging from mild stomach bloating and cramping and diarrhea. Similarly, a total of seven adverse events were reported during PPCBC30 and consisted of upset stomach, low back pain, acidity (belching), and muscle pain. Only the muscle pain reported in PPCBC30 by one participant was reported as moderate severity as all other reported adverse events were mild.

## Discussion

Using a randomized, double-blind, crossover study design, the primary findings from the present study revealed that area under the curve values for three individual amino acids (alanine, tryptophan, and cysteine), essential amino acids, and total amino acids were greater when BC30 was co-ingested with a plant protein concentrate when compared to isolated ingestion of an identical dose of the plant protein concentrate in healthy, older women. Additionally, peak plasma concentrations of tryptophan, cysteine, essential amino acids, and total amino acids were also greater when BC30 was added to a plant protein concentrate.

These findings align with the previous findings of Stecker et al. [8] who reported that arginine and isoleucine area under the curve values were greater in addition to greater peak concentrations for arginine, serine, ornithine, methionine, glutamic acid, phenylalanine, isoleucine, tyrosine, essential amino acids, and total amino acids when BC30 was added to a milk protein concentrate. These findings, however, do contrast with Townsend et al. [16] who had 22 healthy, active men (24.3 ± 3.2 years) supplement with a 25-g dose of whey protein for 14 days in a crossover fashion with and without the addition of a daily one billion CFU dose of *Bacillus subtilis* and reported no changes in amino acid appearance. While the study designs are similar between these two investigations, the key difference was the probiotic strain used in each investigation. In addition to the strain employed, the source of protein and age of participants may have also explained why the outcomes differed between the studies. More to this point, Jäger and colleagues [6] supplemented younger individuals with a 20-g dose of pea protein with and without a combination of different probiotic strains [5 billion CFU L. *paracasei* LP-DG® (CNCM I-1572) plus 5 billion CFU L. *paracasei* LPC-S01 (DSM 26760)] and, in alignment with results from the present study, reported an improvement in amino acid appearance when the probiotic was co-ingested with pea protein.

In the stomach, dietary protein is cleaved into polypeptides by proteases at low pH. Further degradation in the intestine by luminal proteases and membrane bound peptidases results in the formation of peptides and amino acids. Amino acids enter the cell via various amino acids transporters. Di- and tripeptides are translocated via cotransport with protons by the transporter PEPT1. In the cytosol, peptides are mostly degraded leaving the cell as amino acids. Animal proteins contain simple sugars (lactose), while plant proteins contain complex carbohydrates, reducing and slowing down amino acid absorption from plant proteins [4, 23]. The ability of BC30 to influence protein digestibility and enhance amino acid absorption is linked to the release of proteases, peptidases, and carbohydrases to facilitate protein digestion [4, 30], and to changes in the intestinal microflora improving the absorption of small peptides and amino acids by enhancing the epithelium’s absorption ability [31]. In this respect, findings from the present study further extend the evidence surrounding BC30’s ability to survive the human gut and support previous in vitro work that document BC30’s ability to aid in the breakdown of protein [7, 9] as well as the previous human evidence by Stecker and colleagues. The scope of our analytic approach limits our ability to explain why some amino acids are impacted more than others by the presence of BC30. In this respect, it remains possible that the amino acid structure or other electrochemical aspects may have prevented appropriate interaction with BC30. In addition, and along these lines, BC30 may have exerted selective influence over certain amino acid transporters which may have subsequently impacted the extent to which amino acid transport could occur. While beyond the scope of the present investigation, future research could explore this possibility to better understand how BC30 and other probiotic strains may impact protein digestion and amino acid appearance.

The present study advances knowledge surrounding co-ingestion of probiotics and protein in two ways. First, this investigation used a blend of rice and pea protein concentrate. As discussed throughout recent reviews [4, 17, 23], plant proteins have increased in popularity and are in much greater demand than in previous years. This popularity is in large part to the greater sustainability associated with plant protein production and ingestion as well as many studies which associated favorable health outcomes associated with more plant ingestion [24]. A key challenge, however, associated with plant protein ingestion, particularly when viewed in consideration of optimal muscle and metabolic health are the lower levels of many amino acids in plant versus animal sources [18]. In this respect, results from the present investigation were able to significantly increase the amount of essential amino acids and seemingly somewhat help to overcome the compositional outcomes found in various plant proteins. Further to this point, pea protein is low in cysteine and results from the present investigation significantly increased amount of cysteine found in the collected blood when BC30 was added. Moreover, another challenge commonly highlighted with greater plant protein ingestion is its lower levels of digestibility, which impacts the total amount of protein that needs to be ingested to realize key health outcomes [25]. Collectively, these reasons have fueled hesitance by consumers to embrace plant proteins as viable protein sources to drive desired changes in metabolic and muscle health. In evaluating the outcomes and impact of our previous investigation that demonstrated the ability of BC30 to improve amino acid appearance when co-ingested with milk protein concentrate, a top priority of the present study was to see if this reported action of BC30 was also observed with ingestion of plant proteins. In this respect, many proteins found in plants are bound to complex carbohydrate structures which are highlighted as key explanations why digestion of plant proteins is slower and considered inferior to animal proteins. For these reasons, BC30’s ability to increases enzymes for both protein and carbohydrate digestion makes it an ideal probiotic to facilitate greater digestion of plant proteins.

In addition to using a plant protein source, the current study was one of the first investigations to use a cohort of older adults when examining the potential of a probiotic to impact protein digestion and amino acid absorption. Two key changes relative to protein intake occur with an aging population that challenge one’s ability to get adequate amounts of protein to optimize health and promote well-being. First, previous reports indicate that aging populations typically struggle to consume enough protein both per meal and across an entire 24-h time period [26, 27]. Second, age-related “anabolic resistance” is a well-established phenomenon [28] which leads to a situation where larger doses of protein are needed to stimulate rates of muscle protein synthesis. In addition, age-related declines in digestive enzymes in elderly people have been observed, which further challenges an elderly person’s ability to sufficiently digest protein consumed in the diet. Additional investigations are needed to establish the physiological and health implications of increasing amino acid delivery in aged populations. When viewed together, these considerations demonstrate the need for various interventions to help increase amino acid delivery to key tissues of older individuals and helped form the primary basis of this study’s rationale. All told, the results from the study demonstrate the ability of BC30 to increase amino acid appearance from a standardized dose of plant protein concentrate which may effectively help to reduce the protein dose required to be efficacious.

Beyond the key study rationale considerations previously highlighted (incorporation of plant protein source and an older study cohort), a key strength of this investigation was the randomized, double-blind, crossover study design with an isocaloric and isonitrogenous control group. Strict control was maintained to exercise and diet considerations prior to each study visit and the 2-week supplementation period used as part of this study aligned directly with other studies of this nature [6, 29]. Compliance to the supplementation protocol was very high (four total doses missed across all study participants, ~ 99%, in both conditions). All collected blood samples were processed and analyzed under identical conditions. A key limitation from our project was our lack of mechanistic inquiry into the observed changes. In this respect, the collection of urine or fecal samples would have increased our understanding of how much protein was assimilated throughout each condition. Relevant follow-up work could include the collection of skeletal muscle tissue to better understand the impact of BC30 ingestion on muscle or whole-body protein synthesis. Additional follow-up research is necessary to understand to what extent the combination of BC30 with protein ingestion can impact associated health or performance outcomes seen from several weeks of BC30 and protein co-ingestion.

## Conclusion

In concert with previous findings in a younger mixed gender cohort with milk protein, the addition of BC30 to a daily 20-g dose of plant protein concentrates in healthy older women improved AUC and *C*_*Max*_ values in several individual amino acids and amino acid combinations. These results provide additional evidence that adding specific probiotic strains such as BC30 to various forms of protein can improve the appearance of amino acids in the blood. These outcomes hold great relevance to any population who is challenged to consume adequate doses of protein such as the aged or any population with gastrointestinal compromise that may lack the digestive efficiency required to assimilate larger doses of protein.

## Data Availability

The datasets used and/or analyzed during the current study are available from the corresponding author upon reasonable request.

## Abbreviations

yr: Year
cm: Centimeter
kg: Kilogram
g: Gram
mL: Milliliters
L: Liter
BIA: Bioelectrical impedance analysis
°C: Degrees celsius
rpm: Revolutions per minute
BCAA: Branched chain amino acids
EAA: Essential amino acids
AA: Amino acids
AUC: Area under the curve
*C*_*Max*_: Maximum amino acid concentration
*T*_*Max*_: Measured time point for maximum amino acid concentration
µmol-h/L: Millimoles an hour per liter
µmol/L: Millimoles per liter
CONSORT: Consolidated Standards of Reporting Trials
